# Immunostimulating RNA Delivered by P1500 PEGylated Cationic Liposomes Limits Influenza Infection in C57Bl/6 Mice

**DOI:** 10.3390/pharmaceutics12090875

**Published:** 2020-09-14

**Authors:** Elena P. Goncharova, Aleksandra V. Sen‘kova, Innokenty A. Savin, Tat‘yana O. Kabilova, Marina A. Zenkova, Valentin V. Vlassov, Elena L. Chernolovskaya

**Affiliations:** Institute of Chemical Biology and Fundamental Medicine SB RAS, 630090 Novosibirsk, Russia; egn@niboch.nsc.ru (E.P.G.); alsenko@mail.ru (A.V.S.); kesha_savin@mail.ru (I.A.S.); kabilova@niboch.nsc.ru (T.O.K.); marzen@niboch.nsc.ru (M.A.Z.); vvlassov@mail.ru (V.V.V.)

**Keywords:** immunostimulating RNA, influenza a virus, PEGylated cationic liposomes, IFN-α, innate immunity

## Abstract

The emergence of highly pathogenic viruses and a high speed of infection spread put forward the problem of the development of novel antivirals and their delivery vehicles. In this study, we investigated the antiviral effect of the previously identified immunostimulatory 19-bp dsRNA (isRNA) with 3′-nucleotide overhangs, which stimulates interferon α synthesis when delivered using cationic liposomes consisting of 1,26-bis(cholest-5-en-3β-yloxycarbonylamino)-7,11,16,20-tetraazahexacosan tetrahydrochloride and lipid-helper dioleoylphosphatidylethanolamine and its PEGylated formulation P1500 in vitro and in vivo. In vitro data showed that isRNA/2X3-DOPE complexes protected L929 cells from encephalomyocarditis virus infection, while isRNA/P1500 complexes were not active, which correlates with their lower transfection activity in cell culture. Comparison of the interferon-inducing activity of isRNA in BALB/c, CBA and C57Bl/6 mice showed that PEGylated liposomes significantly enhance the interferon-inducing activity of isRNA in vivo. The antiviral efficacy of the isRNA in vivo was considerably affected by the delivery system. The cationic liposomes 2X3-DOPE did not enhance the antiviral properties of isRNA in vivo. Similar liposomes equipped with a PEGylated lipoconjugate provided a pronounced anti-influenza effect of the isRNA in vivo. Administration of isRNA to C57Bl/6 led to a decrease in virus titers in the lungs and a significant decrease in the severity of the infection. Administration of a similar formulation to BALB/c mice caused only a mild antiviral effect at the initial stages of the infection. The data show that isRNA in combination with the PEGylated delivery system can be considered an effective means of suppressing influenza A infection.

## 1. Introduction

The emergence of highly pathogenic viruses and the re-emergence of previously controlled infections, as well as drug resistance and the accelerated spread of infectious agents as a result of travel and transportation, make the development of new methods of combating viral diseases a top priority. Antiviral agents can be divided into the following two groups: highly specific compounds that target a specific step of the virus life cycle (i.e., inhibitors of viral enzymes) [1] and compounds with different modes of action that either interact with viral components and cause virus inactivation [2,3,4] or activate the host immune system [5]. The first type of agents is commonly used in modern antiviral therapy, and their clear advantage is their high specificity for target viruses [6]. At the same time, the use of these agents is hindered by the high probability of mutations in the genome of some viruses (influenza virus, HIV, etc.), which leads to the resistance of these viruses to the therapeutic agents [7]. Despite all efforts, specific therapeutic agents were found for only a small number of viruses [8].

Thus, finding new antiviral drugs against the influenza virus is still an urgent problem. Currently, researchers are focused on immunotherapeutic drugs, eliminating the need to develop new specific drugs for each virus type or viral strand that directly affect the replication of the influenza virus. Activation of the innate immune system by appropriate immunotherapeutics occurs in the same way as during infection with the influenza virus and leads to the synthesis of a number of cytokines involved in the development of an antiviral response and the formation of an antiviral state that prevents the spread of infection between body cells [9]. Type I interferons (IFNs) play a central role in the development of the antiviral response, inducing resistance of uninfected cells to infection and determining the possibility of a successful outcome of the disease. Type I IFNs stimulate the secretion of a number of cytokines, such as IL-10 and IL-6, and block the synthesis or functions of others, such as IL-17, IL-1 and IFN-γ [10]. However, the use of interferon preparations is limited by their pyrogenicity and allergenicity, the risk of autoimmune processes, and the need for repeated administration of a daily dose [11]. Inducers of an endogenous interferon, including dsRNA, are devoid of these shortcomings. The interferonogenic activity of dsRNA preparations has been intensively studied in order to create therapeutics for the treatment of viral diseases and to prevent the progression of tumors [12].

Earlier, we identified immunostimulatory 19-bp dsRNA (isRNA) with 3′-nucleotide overhangs, which stimulates IFN-α synthesis and suppresses the growth and metastasis of tumors [13,14,15,16]. Since isRNA stimulates IFN-α synthesis, which is an important component of antiviral protection, it is reasonable to assess the antiviral potential of isRNA against influenza virus.

We have previously developed an efficient liposomal nucleic acid delivery system based on polycationic amphiphile 1,26-bis (cholest-5-en-3-yloxycarbonylamino) -7,11,16,20-tetraazagehexacosane tetrahydrochloride (2X3) and lipid-helper dioleoylphosphatidylethanolamine (DOPE), which efficiently delivers nucleic acids in vitro and in vivo [17,18,19]. We synthesized PEG-containing lipoconjugates that differed in the molecular weight of PEG (800, 1500 and 2000 kDa) and showed that increasing the length of PEG in the lipoconjugate decreased the effectiveness of the delivery of nucleic acids by the liposomes containing these lipoconjugates in vitro, but improved biodistribution, duration of circulation in the bloodstream and interferon-inducing effect of isRNA in vivo [20]. IsRNA exhibited the most pronounced interferonogenic effect in combination with liposomes containing 1500 kDa PEG (P1500); therefore, in the present study, we chose this formulation and 2X3-DOPE core system to study the antiviral effect of isRNA.

In this study, we evaluated the antiviral effect of isRNA on mouse strains differing in the ability to activate IFN-α synthesis in response to interferon inducers and the effect of the delivery system on the antiviral effect of isRNA. We found that isRNA in combination with PEG-containing cholesterol-based cationic liposomes stimulates the synthesis of IFN-α in mice and efficiently interferes with influenza infection.

## 2. Materials and Methods

### 2.1. Synthesis of isRNA

Oligoribonucleotides (strand 1: 5′-GUGUCAGGCUUUCAGAUUUUUU-3′; strand 2: 5′-AAAUCUGAAAGCCUGACACUUA-3′) were synthesized, isolated and annealed as described previously [20].

### 2.2. Cell Cultures

Madin-Darby canine kidney (MDCK) epithelial cells and L929 murine fibroblast were purchased from the Bank of Cell Cultures (Institute of Cytology, Saint-Petersburg, Russia). The cells were maintained in Dulbecco’s modified Eagle medium (DMEM; Sigma-Aldrich, St. Louis, MO, USA) supplemented with 5% fetal bovine serum (FBS; Sigma-Aldrich, St. Louis, MO, USA), 100 units/L penicillin, 100 mg/mL streptomycin and 0.25 mg/mL amphotericin (antibiotic-antimycotic solution, Sigma-Aldrich, St. Louis, MO, USA) at 37 °C in a humidified atmosphere containing 5% CO_2_.

### 2.3. Animal Studies

Inbred female BALB/c and C57Bl/6 mice at the age of 6–8 weeks with an average weight of 16–18 g were obtained from the State Research Center of Virology and Biotechnology VECTOR (Koltsovo, Russia). Eight- to ten-week-old male CBA/LacSto (CBA) mice with an average weight of 23–27 g were obtained from the vivarium of the Institute of Cytology and Genetics SB RAS. The mice were quarantined for 72 h prior to experimental manipulation. Animals were kept in the vivarium of the Institute of Chemical Biology and Fundamental Medicine, SB RAS, with a natural light regime on a standard diet for laboratory animals (GOST (State Standard) R 5025892) in compliance with the international recommendations of the European Convention for the Protection of vertebrate animals used for experimental studies (1997) and the rules of laboratory practice in the performance of preclinical studies in the Russian State Standards (R 51000.3-96 and 51000.4-96). The experimental protocols were approved by the Committee on the Ethics of Animal Experiments with the Institute of Cytology and Genetics of SB RAS (protocol #22.11 from 30 May 2014).

Mice (*n* = 6) were treated via intravenous (i.v.) injection through the tail vein with isRNA (0.5 µg/g) precomplexed with cationic liposomes at an N/P ratio of 6/1 six hours prior to infection with influenza A/WSN/33 (H1N1; hereafter named IVA). For the delivery of isRNA cationic liposomes 2X3-DOPE [17] and PEG-containing cationic liposomes P1500 [20] were used. Complexes of cationic liposomes with isRNA were prepared in a serum-free Opti-MEM medium by mixing equal volumes of isRNA and liposomes, followed by incubation for 20 min at room temperature. Particle size and zeta potential was measured using Zetasizer Nano ZS (Malvern Instruments, Malvern, UK). MOCK-treated (treated the same way except that no isRNA was added) mice received the same volume of liposomes in Opti-MEM.

Commercially available Cycloferon (N-methylglucamine 2-(9-oxoacridin-10(9H)-yl) acetate, STPF POLYSAN, St. Petersburg, Russia) was prepared as a solution in Opti-MEM, and mice were i.v. treated with cycloferon (90 mg/kg) 6 h prior to challenging with IVA as a positive control. IVA infected mice receiving no treatment were used as a negative control. Six-hour post-injection mice were anesthetized by intraperitoneal injection of tribromoethanol (Avertin^®^) at a dose of 250 mg/kg and then infected intranasally with 0.8 LD_50_ (the amount of virus that is sufficient to kill 50 percent of mice) of IVA in 40 µL of PBS (unless otherwise specified). Detection of viral titers was carried out on days 1 and 3, and histopathological analysis was carried out on days 3, 7 and 14 post-infection (p.i.) in the case of BALB/c mice. Detection of viral titers and histopathological and immunohistochemical analysis were carried out on days 1, 3 and 5 p.i. in the case of C57Bl/6 mice. The lungs of mice were collected and weighed at a specified time. DMEM supplemented with antibiotic–antimycotic solution and 2 μg/mL *N*-p-Tosyl-l-phenylalanine chloromethyl ketone (TPCK)-treated trypsin (infection medium) was added to lungs at a ratio of 1:10 (*v*/*v*) of lung tissue to the medium. Homogenates were prepared using a Sonopuls HD 2070 Ultrasonic Homogenizer (Bandelin, Berlin, Germany). The viral titers were determined using the focus forming assay (FFA).

### 2.4. Focus Forming Assay (FFA)

Influenza virus titers were determined by the FFA in MDCK cells. Lung homogenates were serially diluted 10-fold with an infection medium and incubated with MDCK cells for 24 h at 37 °C with 5% CO_2_. Thereafter, MDCK monolayers were washed twice with PBS and subsequently fixed with ice-cold 80% acetone for 15 min at room temperature. Viral foci were stained using a mouse monoclonal antibody against influenza A NP (nuclear protein), 1/2500 (MAB8258, Millipore, Burlington, MA, USA), secondary antibody anti-mouse IgG biotin conjugate, 1/4000 (B7151, Sigma-Aldrich, St. Louis, MO, USA), peroxidase labeled streptavidin, 1/4000 (Sigma-Aldrich S2438, USA) and 3-amino-9-ethylcarbazole (AEC; Sigma-Aldrich, St. Louis, MO, USA). Focus-forming units (FFU; NP-positive red-colored cells located apart from one another at a distance of two uncolored cells) were then calculated, and viral titers were expressed as FFU per mL.

### 2.5. Interferon Alpha/Beta Bioassay

To characterize the type I IFN-inducing activity of isRNA, the level of biologically active IFN-α/β was measured by the standard IFN assay using the model system composed of L929 murine fibroblast cells and mice encephalomyocarditis virus (EMCV) as the challenge virus. Briefly, L929 cells were seeded in 96-well plates at a density of 1 × 10^4^ per well and incubated for 24 h. isRNA (100 nM) precomplexed with 2X3-DOPE or P1500 liposomes, as described above, was added to the wells (*n* = 8) of the 96-well plate and incubated for 6 h at 37 °C. Then, EMCV at a dose of 100 50% tissue culture infective doses (TCID_50_) was added to each well and incubated for 24 h at 37 °C. Infected cells treated with cycloferon, the reference interferon inducer, at a concentration of 9.8 µM in a well were used as a positive control. Infected cells incubated without treatment were used as a negative control. The cytopathogenic effect of EMCV was estimated using the МТТ (3-[4,5-dimethylthiazol-2-yl]-2,5-diphenyltetrazolium bromide) test. The protective activity of isRNA or cycloferon was calculated as the percent inhibition of the cytopathogenic effect of the virus relative to the uninfected cells.

### 2.6. IFN-α2 ELISA

BALB/c, CBA/LacSto and C57Bl/6 mice were i.v. injected with isRNA (0.5 µg/g) precomplexed with cationic liposomes (N/P = 6/1), and blood was collected 6 h after injection via a head clipping. The serum was prepared from the whole blood by coagulation for 30 min at 37 °C and subsequent centrifugation. The concentration of IFN-α2 in the serum was determined with the Antibody Pair kit (Abcam, San Jose, CA, USA) in accordance with the manufacturer’s instructions. The cytokine level was measured in duplicate in individual serum samples from three mice per group.

### 2.7. Complete Blood Count and Toxicity

The systemic toxicity of IVA and the studied compounds in the mice was evaluated during the experiments by determination of the body weight and blood formula. Mice were anesthetized by intraperitoneal injection of tribromoethanol (Avertin^®^) at a dose of 250 mg/kg, and blood samples were withdrawn by retro-orbital sinus puncture using heparinized microcapillary tubes. Then, hematological parameters, which included leukocytes with leukocyte differential count, erythrocytes and hemoglobin, were estimated using a hematology analyzer (MicroCC20Vet, High Technology Inc., North Attleborough, MA, USA).

### 2.8. Histology and Immunohistochemistry

For the histological study, lung specimens from each animal were collected during autopsy and fixed in 10% neutral-buffered formalin (BioVitrum, St. Petersburg, Russia), dehydrated in ascending ethanols and xylols, and embedded in HISTOMIX paraffin (BioVitrum, St. Petersburg, Russia). Paraffin sections (5 μm) were sliced on a Microm HM 355S microtome (Thermo Fisher Scientific, Waltham, MA, USA) and stained with hematoxylin and eosin, microscopically examined, and scanned using an Axiostar Plus microscope equipped with an Axiocam MRc5 digital camera (Zeiss, Ulm, Germany) at magnification of ×100 (hematoxylin and eosin staining) and ×400 (immunohistochemistry).

Lung sections for immunohistochemical studies (3–4 μm) were deparaffinated and rehydrated. Antigen retrieval was carried out after exposure in a microwave oven at 700 W. The samples were incubated with Anti-TNF-α (ab1793, Abcam, San Jose, CA, USA) and Anti-lysozyme (ab182422, Abcam, San Jose, CA, USA) specific monoclonal antibodies according to the manufacture’s protocol. Next, the sections were incubated with secondary HPR-conjugated antibodies (Spring Bioscience detection system, Spring Bioscience, Pleasanton, CA, USA), exposed to the DAB substrate and stained with Mayer’s hematoxylin.

Morphometric analysis of lung sections was performed by point counting using a counting grid that consisted of 100 testing points in a testing area equal to 3.2 × 10^6^ μm^2^. Ten to fifteen random fields were studied in each specimen, forming 60–90 fields for each group of mice in total. Morphometric analysis of lungs included evaluation of the volume densities (Vv, %) of normal lung tissue, lung tissue with inflammatory infiltration, lung tissue with proliferative changes and volume densities (Vv, %) of blood vessels and bronchi as part of lung parenchyma. Numerical densities (Nv) of lysozyme-positive cells and TNF-α-positive cells were also evaluated.

The volume density (Vv, %) of the studied histological structure representing the volume fraction of tissue occupied by this compartment was determined from the points lying over this structure and calculated using the following formula: Vv = (P_structure_/P_test_) × 100%, where P_structure_ denotes the number of points over the structure and P_test_ denotes the total number of test points, which was 100 in this case. The numerical density (Nv) of the studied histological structure, indicating the number of particles in the unit tissue volume, was evaluated as the number of particles in the square unit, which was 3.2 × 10^6^ μm^2^ in this case.

### 2.9. Statistical Analysis

Data are expressed as the mean ± SD. Statistical analysis was performed using the two-tailed unpaired *t*-test. *p*-values of less than 0.05 were defined as statistically significant.

## 3. Results

The objective of this work was to evaluate the potential of short double stranded isRNA to combat viral infection, namely infection caused by the influenza virus. It was shown [13] that the antiproliferative activity with respect to tumor cells, as well as activation of cytokine secretion by isRNA, requires the presence of transfection agents. isRNA delivery to the cell, mediated by liposomes, is necessary for the manifestation of the interferon-inducing activity of isRNA, and the effectiveness of both the interferon-inducing and antiproliferative effects depends on the efficiency of isRNA delivery into the cells [20,21]. This indicates that isRNA activates some cytosolic or endosomal sensors, similarly to viral RNA. In the present study, we used two cationic liposome formulations based on the polycationic amphiphile 2X3, which is 1,26-bis(cholest-5-en-3β-yloxycarbonylamino)-7,11,16,20-tetraazahexacosan tetrahydrochloride [17,18]. It was shown that cationic liposomes consisting of 2X3 and lipid-helper dioleoylphosphatidylethanolamine (DOPE; 1:1 mol.) efficiently delivered various nucleic acids, including isRNA, to target cells both in vitro and in vivo [22]. The addition of PEG (MW 1500) lipoconjugate to the 2X3-DOPE formulation (2 mole%) resulted in the formulation P1500 [20], providing prolonged circulation of lipoplexes in the bloodstream and increased efficiency of IFN-α induction by isRNA. Hydrodynamic diameters, polydispersity index (PI) and ξ-potentials of liposomes and lipoplexes formed by isRNA and liposomes (N/P = 6/1) are shown in Table 1.

### 3.1. Interferon-Inducing Activity of isRNA In Vitro

**Figure 1:**
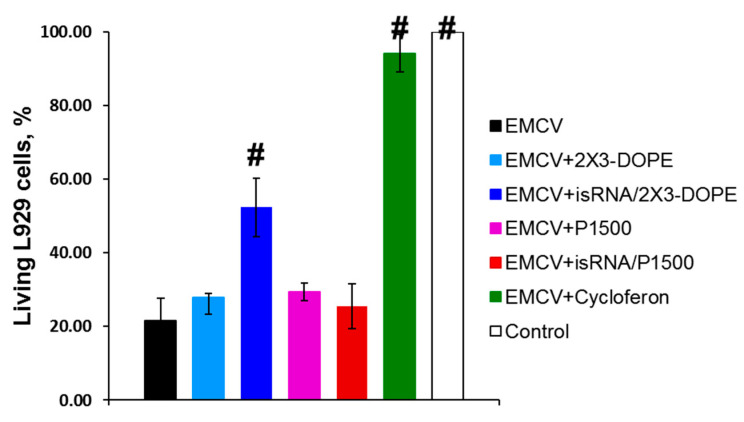
The antiviral effect of isRNA on the model of the mice encephalomyocarditis virus (EMCV) and mouse fibroblastic cell line L929. # Statistically significant difference relative to isRNA/2X3-DOPE treated mice, *p* ≤ 0.05.

In the first stage of the study, we evaluated the in vitro interferon-inducing activity of isRNA by measuring the protective effect of IFN induced by isRNA using a model system of the mouse fibroblastic cell line L929 infected with EMCV [23]. It has been shown that IFN protects L929 from EMCV-induced cell death through inhibiting viral replication [24]. This approach makes it possible to assess the cumulative antiviral action of all interferon subtypes secreted by the cell in response to an immunostimulating stimulus. In this experiment, cycloferon, a known pharmacological interferon inducer, was used as a positive control. This model system is widely used to assess the efficiency of IFN induction by various chemical compounds [25]. L929 cells were treated with isRNA precomplexed with cationic liposomes 2X3-DOPE or P1500 or with a control inducer. Then, after 6 h the cells were infected with EMCV at a dose of 100 TCID_50_. The cytopathic effect of the virus was evaluated 24 h post infection using the MTT test. Treatment of L929 cells with cycloferon entirely protected the cells (94% cells were viable) from the cytotoxic effect of the virus (Figure 1). The isRNA/2X3-DOPE complex protected 57% of cells in this population, while isRNA/P1500 complexes were not active since only 26% of the cells remained viable, which is similar to untreated and MOCK-treated controls (21% viable cells). These results correlate well with our data on the ability of 2X3-DOPE and P1500 formulations to mediate the delivery of plasmid DNA and siRNA into the cultured cells [20].

Apparently, the most important factor in the in vitro experiments is the efficiency of isRNA delivery into cells, which is highest for 2X3-DOPE liposomes [20]. Thus, the data obtained indicates that treatment of cells with isRNA/2X3-DOPE markedly reduced viral infection in the cell line.

### 3.2. Interferon-Inducing Activity of isRNA In Vivo

**Figure 2:**
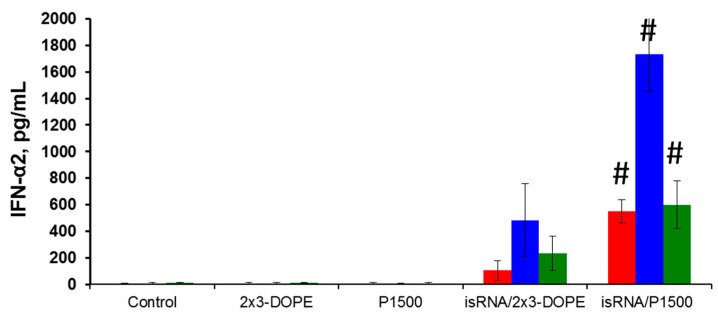
The level of INF-α2 in the blood serum of BALB/c (red bars), CBA (blue bars) and C57Bl/6 (green bars) mice after i.v. administration of isRNA/2X3-DOPE or isRNA/P1500 complexes at a dose 0.5 µg/g. # Statistically significant difference relative to isRNA/2X3-DOPE treated mice, *p* ≤ 0.05.

We compared isRNA-mediated IFN-α2 induction in different mouse strains in order to identify the mice strain convenient for testing the antiviral effects of isRNA. Models of influenza infection in BALB/c and C57Bl/6 mice are widely used and are well described in the literature; therefore, we used them in our experiments. CBA mice that previously demonstrated induction of IFN-α synthesis after i.v. injection of isRNA in combination with the transfection agent [16] were used as a positive control. The data showed that the IFN-α2 levels in blood serum 6 h after i.v. administration of isRNA/2X3-DOPE or isRNA/P1500 complexes varied considerably among the strains (Figure 2). Comparison of the interferon-inducing activity of isRNA in BALB/c, CBA and C57Bl/6 mice showed that PEGylated liposomes significantly enhance the interferon-inducing activity of isRNA in vivo. The most significant increase in the serum IFN-α2 level after isRNA/2X3-DOPE administration was detected in CBA mice (up to 500 pg/mL), whereas the level of IFN-α2 in C57Bl/6 mice was about 50% lower (up to 230 ng/mL). The IFN-α2 level in BALB/c mice was much lower and did not exceed 105 pg/mL. Injection of isRNA/P1500 caused a more significant induction of IFN-α2 synthesis: its serum levels were 550, 1733 and 600 pg/mL for BALB/c, CBA and C57Bl/6 mice, respectively (Figure 2). The data obtained by us previously showed that liposomes with long PEG-linkers (P1500) significantly (up to three times) increase the immunostimulating effect of isRNA in CBA mice as compared with isRNA/2X3-DOPE complexes without activation of the synthesis of tumor necrosis factor [20]. The data obtained in the current study confirmed the key influence of the delivery system on the effectiveness of the interferonogenic action of isRNA. In in vivo experiments, the circulation time and stability of the complexes in serum play important roles.

Taking into account the importance of interferons for protection against viral infection, we selected BALB/c and C57Bl/6 mice for evaluation of the antiviral potential of isRNA on a model of influenza infection and determined the dependence of antiviral activity on the activation of IFN-α secretion.

### 3.3. Antiviral Effect of isRNA In Vivo

**Figure 3:**
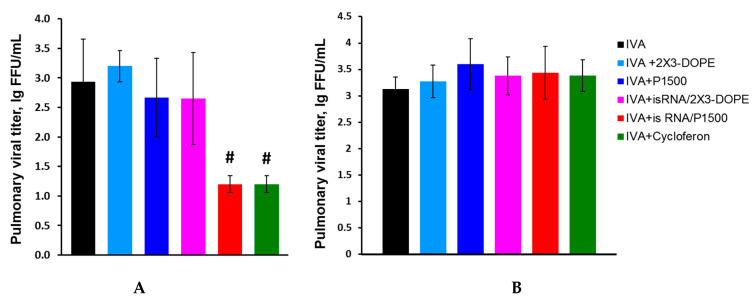
Effect of interferon inducers on the pulmonary viral titer in IVA infected C57Bl/6 (**A**) and BALB/c (**B**) mice 24 h after infection with IVA at a dose of 0.8 LD_50_. # Statistically significant difference relative to untreated IVA-infected BALB/c mice, *p* ≤ 0.05.

We compared the course of influenza infection in BALB/c and C57Bl/6 mice to determine the efficacy and mechanism of the antiviral action of isRNA. Mice were intranasally infected with IVA (0.8 LD_50_). isRNA precomplexed with cationic liposomes 2X3-DOPE or P1500 was administered intravenously (i.v.) at a dose of 0.5 μg/g of the mice weight 6 h prior to infection. MOCK-treated mice received the same amount of cationic liposomes. IVA-infected mice without treatment were used as a control. Pulmonary viral titers in C57Bl/6 mice 24 h after infection were significantly reduced only in the groups treated with isRNA/P1500 and cycloferon (control interferon inducer), but were similar in groups of mice treated with isRNA/2X3-DOPE, MOCK-treated and untreated mice (Figure 3A).

The reduction in virus titer observed after administration of isRNA/P1500 complexes indicates that the PEGylated liposomes enhanced the immunostimulating activity of isRNA and accordingly increased its antiviral action. A different result was observed in BALB/c mice. Neither isRNA complexes with liposomes nor cycloferon influenced the development of the infection; pulmonary virus titers were similar in all groups (Figure 3B). For a more detailed assessment of the antiviral effect of isRNA in mice with different responsiveness to isRNA (BALB/c and C57Bl/6 mice), the pathomorphological features of the infectious process were studied.

### 3.4. Effect of isRNA on the Development of IVA Infection in BALB/c Mice

**Figure 4:**
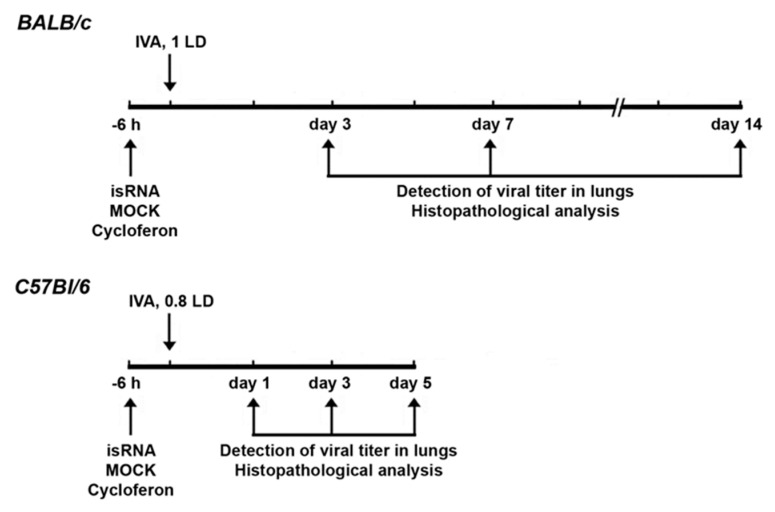
Scheme of in vivo experiments. BALB/c and C57Bl/6 mice were infected intranasally (i.n.) with influenza A virus (IVA) with 1 and 0.8 LD, respectively. isRNA (0.5 µg/g) precomplexed with P1500 liposomes was intravenously (i.v.) administrated to mice 6 h prior to infection. Cycloferon was used as a reference drug with proved immunostimulating activity. Animals were euthanized on days 3, 7 and 14 post infection (p.i.) in the case of BALB/c mice and on days 1, 3 and 5 p.i. in the case of C57Bl/6 mice. Lung tissues were collected for detection of viral titer and histopathological analysis.

isRNA (0.5 µg/g) precomplexed with P1500 liposomes was i.v. administrated to BALB/c mice 6 h prior to infection (Figure 4). Since we observed no changes in the viral titer at 24 h p.i. (Figure 3B) in IVA-infected BALB/c mice upon treatment, in this experiment histopathological analysis was carried out on days 3, 7 and 14 p.i.

**Figure 5:**
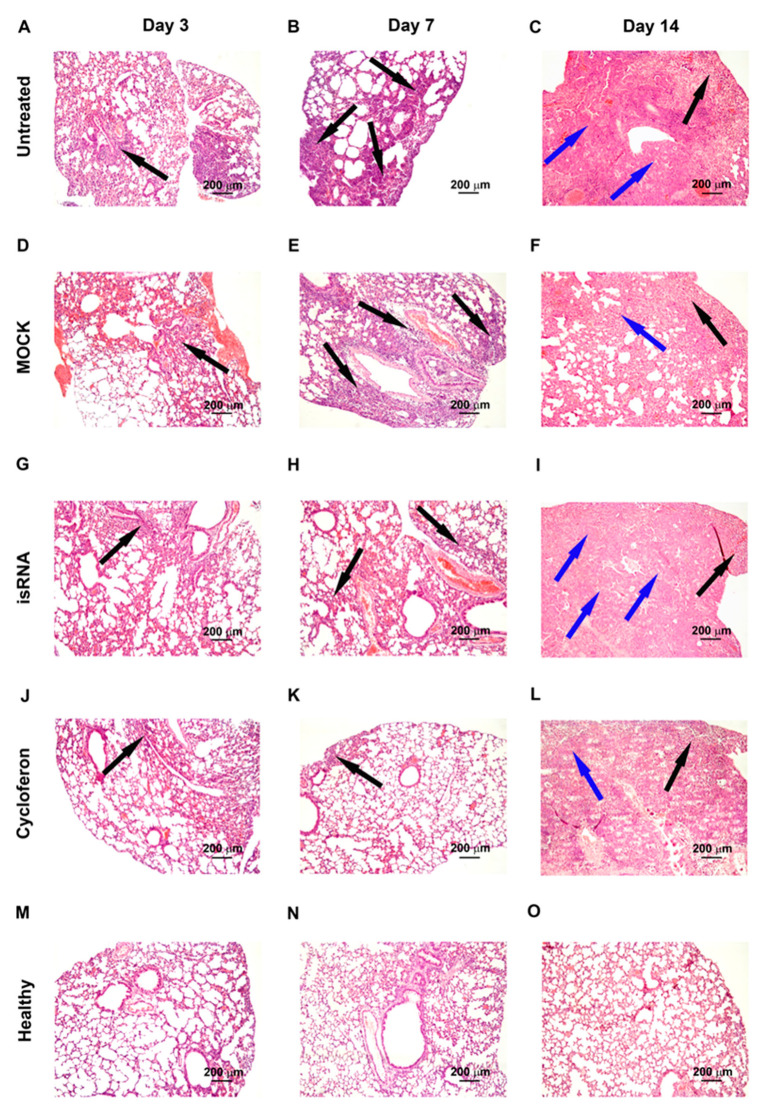
Pathomorphological changes in the lungs of IVA infected BALB/c mice on days 3, 7 and 14 p.i.: untreated (**A**–**C**), MOCK (**D**–**F**), isRNA (**G**–**I**) and cycloferon (**J**–**L**) treated in comparison with healthy animals (**M**–**O**). Hematoxylin and eosin staining. Original magnification ×100. Black arrows indicate inflammatory infiltration in the lung tissue. Blue arrows indicate adenomatous structures in the lung tissue.

IVA infection led to significant inflammatory, destructive and discirculatory lung tissue damage associated with the development of severe viral pneumonia (Figure 5). Pathological changes in the lungs were presented by interstitial neutrophil-dominant inflammatory infiltration with the interstitial and alveolar edema and hemorrhage. Inflammatory cellular infiltration was localized predominantly around small blood vessels and bronchioles. Alveoli were flooded with edema fluid mixed with fibrin, erythrocytes and inflammatory cells, presented predominantly by neutrophils with an admixture of lymphocytes and macrophages. The revealed pathological changes in the lungs caused by IVA infection progressed during the experiment and reached 50–60% of the entire lung parenchyma on day 14 p.i. (Table 2, Figure 5).

In addition, at the late stage of IVA infection, proliferative changes in the lungs of BALB/c mice were found. These changes occupied 3–11% of the lung parenchyma and were expressed in hyperplasia and bronchiolar metaplasia of the alveolar epithelium with the formation of adenomatous structures surrounded by the inflammatory infiltration predominately represented by lymphocytes and macrophages (Table 2, Figure 5). Furthermore, a progressive increase in the volume density of blood vessels and a decrease in the volume density of the bronchi during the progression of viral pneumonia are noteworthy and indicate a disturbance of blood circulation and organ architecture.

The described pathological changes in the IVA-infected lung tissue were also observed in the MOCK-treated group.

Administration of isRNA precomplexed with cationic liposomes resulted in moderate protection of IVA-damaged lung tissue in BALB/c mice at the initial stage of infection. The effect of isRNA was similar to the reference drug cycloferon (Table 2, Figure 5). However, with progression of the infection, the protective effect of immunostimulating drugs gradually decreased and pathological changes in the lungs of isRNA- and cycloferon-treated mice were similar to untreated animals (Table 2, Figure 5).

Thus, we could observe a moderate protective effect of isRNA and cycloferon at the initial stage of the infection and its disappearance at the late stage of IVA-induced lung inflammation in BALB/c mice.

### 3.5. Effect of isRNA on the Development of IVA Infection in C57Bl/6 Mice

Since noticeable differences between the experimental groups were observed at the early stages of the development of influenza infection 24 h p.i., we changed the experimental design for C57Bl/6 mice to study the initial effects in more detail (Figure 4). The experiment was carried out in a similar manner as with BALB/c mice.

Analysis of the antiviral effect of isRNA/P1500 in the influenza model on C57Bl/6 mice showed that the titer of the virus was remarkably reduced in the lungs of mice receiving isRNA/P1500 only 24 h after the challenge (Figure 3A, Table 3). However, at 72 h and 120 h p.i., no significant decrease in the titer of the virus in the lungs was revealed (Table 3). To verify the protective effect of isRNA with regard to IVA infection, the pathomorphological features of the infectious process in C57Bl/6 mice was studied on days 1, 3 and 5 p.i.

**Figure 6:**
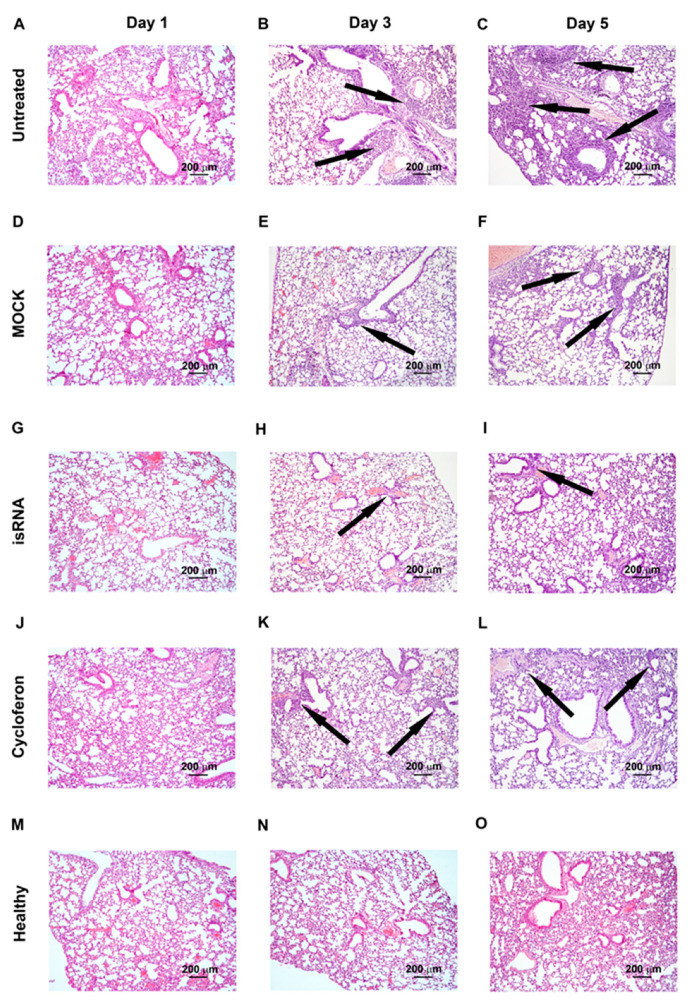
Pathomorphological changes in the lungs of IVA infected C57Bl/6 mice on days 1, 3 and 5 p.i.: untreated (**A**–**C**), MOCK (**D**–**F**), isRNA (**G**–**I**) and cycloferon (**J**–**L**) treated in comparison with healthy animals (**M**–**O**). Hematoxylin and eosin staining. Original magnification ×100. Black arrows indicate inflammatory infiltration in the lung tissue.

IVA infection led to the development of pathological changes in the lung tissue of C57Bl/6 mice, similar to BALB/c mice, reflecting the evolution of viral pneumonia, but expressed to a lesser extent (Table 3, Figure 6). Over the course of the development of the infection, a progressive increase in the inflammatory, destructive and discirculatory changes in the lungs of untreated and MOCK-treated mice was observed, and this amounted to about 20% of the entire pulmonary parenchyma by day 5 p.i. (Table 3, Figure 6). It should be noted that only a slight enlargement of blood vessels by day 5 p.i. was revealed. The volume density of bronchi did not significantly change during the development of the infection in C57Bl/6 mice (Table 3).

Administration of isRNA resulted in significant protection of IVA-damaged lung tissue, comparable to the cycloferon drug with proven immunostimulatory and antiviral activity (Table 3, Figure 6).

The volume density of normal lung tissue of isRNA- and cycloferon-treated mice was approximately 85–90% during the experiment and was similar to the healthy animals. The volume density of inflammatory infiltration in the lungs of isRNA- and cycloferon-treated mice did not exceed 5% of the entire lung parenchyma even by the day 5 p.i.

During evaluation of the amount of vessels and bronchi in the lung tissue of isRNA- and cycloferon-treated mice, only a minor increase in the volume density of blood vessels on day 5 p.i. was revealed, which can indicate a slight plethora that accompanies viral damage of the lungs (Table 3). The volume density of the bronchi did not change or it increased slightly during the experiment, which indicates the absence of impairment of lung architecture in isRNA and cycloferon-treated mice.

Thus, we observed a pronounced protective effect of isRNA and cycloferon at all stages of IVA-induced lung inflammation in C57Bl/6 mice. We can assume that the main mechanism of the antiviral effect of isRNA and cycloferon was the induction of endogenous interferon in C57Bl/6 mice, in which the interferon is synthesized at a high level after induction by different stimuli [26].

### 3.6. Immunohistochemistry

**Figure 7:**
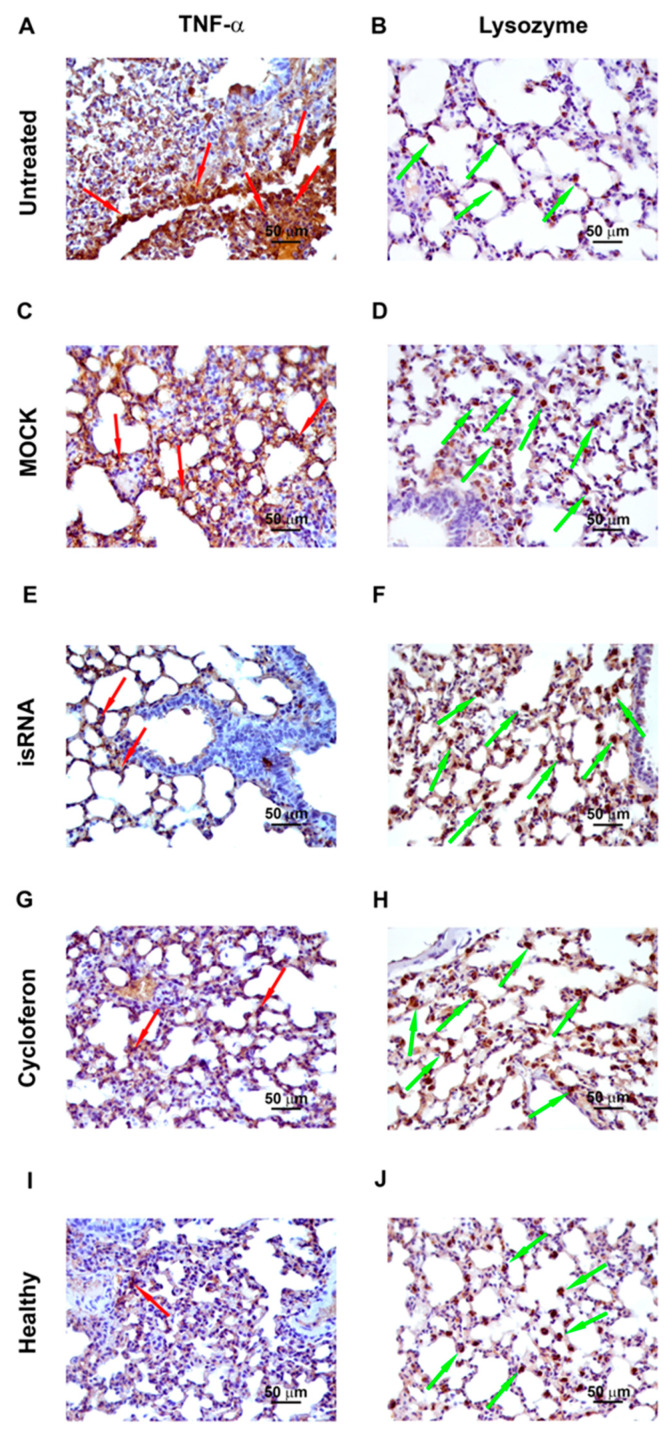
Immunohistochemical staining of TNF-α and lysozyme positive cells in the lung tissue of IVA infected mice on day 5 p.i.: untreated (**A**,**B**), MOCK (**C**,**D**), isRNA (**E**,**F**) and cycloferon (**G**,**H**) treated in comparison with healthy animals (**I**,**J**). Original magnification ×400. Red arrows indicate TNF-α positive areas. Green arrows indicate lysozyme positive cells.

In order to evaluate the expression of proinflammatory cytokine TNF-α and a factor of nonspecific resistance, a lysozyme, immunohistochemical staining with anti-lysozyme and anti-TNFα monoclonal antibodies of the lung tissue sections of IVA-infected C57Bl/6 mice on day 5 p.i. was performed (Table 4, Figure 7).

In healthy mice, the volume density of the TNF-α-positive cells was 14% ± 2.6% and the numerical density of the lysozyme-positive cells was 26.3 ± 1. After IVA infection, the volume density of TNFα-positive cells increased 2.3-fold compared to the healthy control and constituted 32.2% ± 4.7%, and the numerical density of lysozyme-positive cells decreased 2.3-fold and constituted 11.1 ± 0.6. After administration of isRNA/P1500 complexes, the volume density of TNFα-positive cells decreased 1.9-fold, and the numerical density of lysozyme-positive cells increased 2.2-fold compared to the untreated control and constituted 16.6% ± 3.2% and 24.7 ± 1.1, respectively, which is not statistically different from the level of healthy animals. The volume density of TNF-α-positive cells and the numerical density of lysozyme-positive cells in MOCK-treated mice also did not differ significantly from the untreated control and constituted 34.2% ± 4.3% and 13.4 ± 0.8, respectively.

Thus, isRNA decreased the expression of the proinflammatory cytokine TNF-α and increased the expression of the nonspecific protection factor lysozyme in the lung tissue of IVA-infected mice.

## 4. Discussion

Type I IFNs play a central role in antiviral responses, causing the death of virus-infected cells and activating interferon stimulating genes (ISGs), making uninfected cells resistant to viral infection. Two cytosolic RIG-like RNA helicases, RIG-I and MDA5, play key roles in type I IFN induction in response to viral infection. Protein kinase R (PKR) also plays an important role in the recognition of viral nucleic acids, resulting in the induction of INF synthesis during the development of viral infection [27]. When immunostimulatory nucleic acids enter the cell before it is infected by the virus, they interact with pattern recognition receptors (PRRs) designed to recognize viral nucleic acids, and by launching regulatory cascades they form an antiviral state before the cell encounters the virus. Therefore, it can be expected that the prophylactic use of immunostimulatory nucleic acids can be effective against viral infection. Immunostimulatory nucleic acids include long and short double-stranded RNAs, single-stranded RNAs containing immunostimulatory sequence motifs, CpG-containing oligodeoxynucleotides, RNA-containing triphosphates (RIG-I activators) and immunomodulatory spherical nucleic acids (SNAs) [28,29].

Since receptors that recognize viral nucleic acids are located inside the cell, immunostimulatory nucleic acids should be delivered inside the cells in order to activate them.

It should be noted that the requirements for an agent that delivers interfering RNAs (siRNAs), plasmid DNA and immunostimulatory nucleic acids in vivo may differ due to the difference between the target compartment and the target cells. Recently, we found that intravenous administration of original 19-bp isRNA [15] stimulates IFN synthesis and leads to effective suppression of tumor growth and metastases [16]. We showed that cationic liposome 2X3-DOPE significantly enhanced the interferonogenic activity of isRNA as compared to the delivery mediated by lipofectamine 2000 [14,30] and the addition of a PEG-containing lipoconjugate to the 2X3-DOPE formulation enhances the interferonogenic effect of isRNA in vivo [20]. These data suggest that the interferonogenic activity of the isRNA preparation in combination with the corresponding delivery system can be used for the prevention and treatment of viral infections.

In this work, we evaluated the antiviral activity of isRNA on two models: EMCV/L929 cells in vitro and mice-adapted IVA in vivo. isRNA was introduced 6 h before the infection because earlier we found that induction of IFNα is transient: the peak increase in the IFN-α level in murine serum was detected 6 h after i.v. administration of isRNA and 18 h later the IFN-α level was reduced to the initial level [15].

The possibility of protection against EMCV as a result of stimulation of INF synthesis was evaluated in vitro after treatment of lung fibroblasts of mice with isRNA complexed with 2X3-DOPE or P1500 liposomes. The protective effect against EMCV was more pronounced when isRNA/2X3-DOPE liposomes were used, while cell treatment with the isRNA/P1500 formulation did not protect them from EMCV infection. We suggest that the low protective activity of isRNAs/P1500 formulation compared to the isRNA/2X3-DOPE is associated with inefficient delivery of isRNAs to the cells, similar to the low efficiency of delivery observed for this formulation for plasmid DNA and siRNAs in cell culture [20]. We showed that the introduction of a PEG lipoconjugate in the formulation, leading to a decrease in the delivery efficiency in vitro, nevertheless contributes to an increase in the circulation time of isRNA in the bloodstream and reduces its non-specific adsorption by serum proteins [20], which could increase the efficiency of in vivo interferon induction. Comparison of the in vivo protective activity of isRNA/P1500 against influenza virus confirmed the promise of using such modifications of the cationic liposome-based delivery system.

We selected two lines of mice BALB/c and C57Bl/6, with a different ability for the development of the Th1- and Th2-type immune response [31], to evaluate the anti-influenza activity of isRNA in vivo, since it has been demonstrated that the balance of Th1/Th2 is important for the outcome of infection [32]. Th1 cells are thought to be important in clearing intracellular pathogens, whereas Th2 cells are usually associated with responses to parasitic infections [31]. These two inbred lines with a non-identical genetic background are widely used in laboratory studies to evaluate the antiviral activity of various drugs. Mice of these two lines carry a defective polymorphic allele affecting exons 9 through 11 [33,34] of the interferon inducible *MX1* gene, encoding a crucial antiviral factor that inhibits IVA replication and limits disease [35]. There are numerous examples in the literature that mice of these two lines differ in their sensitivity to pathogens [26,36] and in the spectrum of cytokines secreted in response to the action of pathogens [37], vaccines, adjuvants and toll-like receptor agonists [38]. These differences are supposed to be associated with different patterns of expression of toll-like and other pattern-recognizing receptors in immune cells and in other tissues of mice of these lines [39]. This choice of models reflects the real situation in the human population, where individuals respond differently to interferon inducers and “responders” and “non-responders” are present [40]. The incidence of non-responders may depend on the genetic background (as in the case of mouse strains) or it may be determined by the current therapy or the presence of immunosuppressive conditions.

Our data showed that the level of INF-α2 induction after intravenous administration of isRNA/2X3-DOPE in C57Bl/6 mice was higher than in BALB/c mice, however, with more effective stimulation with isRNA/P1500 complexes, the levels of INF-α2 were comparable in both mouse strains (Figure 2), which, considering the nature of the inducer itself, suggests that in this case the induction of INF-α may occur via activation of sensors for foreign RNA, such as RIG I and PKR. Despite the fact that isRNA is not a direct analogue of the agonists of these sensors in terms of the presence of already known immunostimulating motifs and markers, it should be noted that the PRRs that bind to double stranded RNAs are quite flexible. Thus, Lee et al. [41] showed that RIG-I might recognize more complex structures within RNA than the 5′-PPP moiety. In addition, Stewart et al. suggested that TLR3 might play a dual role in the recognition of both long and short dsRNA [42].

The analysis of the protective activity of the isRNA preparation in IVA infected mice showed that only C57Bl/6 mice receiving the isRNA preparation in combination with PEG-containing liposomes showed a decrease in the pulmonary virus titer one day after infection compared to the control group, whereas when isRNA/2X3-DOPE formulation was used a decrease in the pulmonary virus titer was not observed (Figure 3). The protective activity of the isRNA delivered using both transfectants as well as cycloferon was not detected in IVA infected BALB/c mice. Such a difference in the antiviral action of isRNA at comparable levels of IFN induction may be associated with a difference in the spectra of cytokines secreted by Th1 and Th2 responders, as well as the properties of M-1 and M-2 macrophages, the activation of which accompanies the immune response of different types. In particular, it was shown that M-1 macrophages produce more nitric oxide than M-2 macrophages when exposed to IFN or LPS [43].

The importance of histomorphological studies in assessing the prospects of using new immunotherapeutic drugs for the treatment and prevention of influenza infections should be noted. The extent of lung tissue damage and other pathomorphological parameters provide more objective assessment of the antiviral properties of new therapeutics compared to the evaluation of their efficacy based only on the degree of reduction in virus reproduction in the lungs. It is known that the development of influenza infection is characterized by extensive inflammation and necrosis of the bronchoalveolar epithelium, perivascular and peribronchial inflammation and pulmonary edema. Despite the rather temporal restriction of the viral load in the lung tissue of C57Bl/6 mice, which was detected only 24 h post infection, there was a significant decrease in the area of inflammatory infiltration in the lungs of mice receiving i.v. isRNA/P1500 throughout the observation time (5 days), comparable to the action of cycloferon. In addition, a decrease in the expression of the key proinflammatory cytokine TNF-α in comparison with the control group and an increase in the synthesis of a lysozyme, a component of non-specific protection, were revealed in this group, which indicates that the use of isRNA is beneficial for reducing lung damage during influenza infection.

In BALB/c mice, a decrease in the intensity of the inflammatory process was also observed, but it was much less pronounced and can be partially caused by nonspecific activation of the immune response by cationic liposomes, as was shown earlier [44], since the effect was observed also in the MOCK group. I.v. administration of isRNA to mice of this line did not lead to a decrease in the viral load in the lungs, indicating that the immunostimulating properties of the formulations are insufficient to obtain a pronounced antiviral effect. It should be noted that in our experiments a single administration of the drug was used since selection of the dosing regimen is particularly important for therapy with interferon inducers in connection with the formation of an interferon refractory state and this represents a separate task. Previously, we experimentally determined the duration of the refractory state of IFNα synthesis to reinduction by isRNA/2X3-DOPE and found that reactivation of IFN-α synthesis occurs at a time interval of 96 h between injections, while the use of shorter time intervals did not result in the activation of IFNα production. Such dynamics of sensitivity to the induction of the interferon by isRNA allows for the development of schemes with multiple administrations, which may enhance the antiviral effect.

Thus, isRNA in combination with the P1500 cationic liposome formulation reduces the severity of influenza infection in C57Bl/6 mice due to efficient induction of INF-α production followed by the inhibition of virus-induced pneumonia. The diseases caused by IVA share many clinical symptoms with other respiratory infections, including diseases caused by SARS-CoV, SARS-CoV-2 and MERS-CoV. For these diseases, as well as for influenza, specific drugs are not available yet; therefore, non-specific activation of innate immunity can be used to protect against infection. The pathological changes observed in these diseases also have common features, but in the case of COVID-19, pneumonia and lung injury occur much more often and have greater severity and clinical significance than in the case of influenza, which puts a problem regarding protecting lung tissue during the development of the infection on the edge. Thereby, both approaches using isRNA might have the potential for treatment of COVID-19.

## Figures and Tables

**Table 1:** Hydrodynamic diameters, polydispersity index (PI) and ξ-potentials of liposomes and lipoplexes formed by isRNA and liposomes (N/P = 6/1).

Formulation	Diameter, nm	Polydispersity Index (PI)	ξ-Potential, mV
2X3-DOPE	68.82 ± 1.65	0.25 ± 0.01	+42.90 ± 1.99
isRNA/2X3-DOPE	75.72 ± 2.53	0.34 ± 0.02	+44.33 ± 2.536
P1500	75.48 ± 8.55	0.29 ± 0.05	+43.17 ± 1.07
isRNA/P1500	93.02 ± 3.06	0.42 ± 0.02	+33.43 ± 0.49

**Table 2:** Morphological changes in the lung tissue of IVA-infected BALB/c mice without treatment and after isRNA administration.

	Vv Normal Lung Tissue, %	Vv Inflammation, %	Vv Proliferation, %	Vv blood Vessels, %	Vv Bronchi, %
Healthy	82.1 ± 2	-	-	5.8 ± 0.8	11.9 ± 2
**Day 3**					
Untreated	71.4 ± 1.5 *	28 ± 1.3	-	2.4 ± 0.2 *	7.9 ± 0.9 *
MOCK	68.6 ± 2.2 *	23.2 ± 2.6 ^#^	-	2.7 ± 0.7 *	5.1 ± 0.8 *^#^
isRNA	67.2 ± 1.6 *^#^	22.9 ± 1.4 ^#^	-	3.2 ± 0.4 *^#^	6.8 ± 0.9 *
Cycloferon	70.1 ± 1.7 *	16 ± 1.5 ^#^	-	5.2 ± 1 ^#^	8.4 ± 1.4
**Day 7**					
Untreated	52.1 ± 3.6 *	36.6 ± 4	-	6.8 ± 0.8	4.2 ± 1.7 *
MOCK	55.1 ± 2 *	30.4 ± 1.9 ^#^	-	6.9 ± 0.7	7.1 ± 1 *^#^
isRNA	62 ± 1.8 *^#^	24.0 ± 2.1 ^#^	-	7.0 ± 0.8	6.6 ± 0.6 *^#^
Cycloferon	66.3 ± 1.9 *^#^	17.2 ± 1.3 ^#^	-	9.3 ± 0.9 *^#^	6.9 ± 1.1 *
**Day 14**					
Untreated	27.3 ± 4 *	51.6 ± 3.3	9.0 ± 1.9	10.2 ± 1.3 *	1.5 ± 0.6 *
MOCK	24.6 ± 2.8 *	58.1 ± 2.4 ^#^	2.9 ± 1.4 ^#^	10.2 ± 1 *	3.9 ± 0.9 *^#^
isRNA	18.8 ± 2.7 *^#^	52.3 ± 2.1	11.3 ± 2.6	14.5 ± 1.2 *^#^	2.7 ± 0.7 *
Cycloferon	19.8 ± 2.4 *^#^	62 ± 1.9 ^#^	3.3 ± 1 ^#^	9.6 ± 1.1 *	4.5 ± 0.6 *^#^

* Statistically significant difference relative to healthy BALB/c mice, *p* ≤ 0.05. ^#^ Statistically significant difference relative to untreated IVA-infected BALB/c mice, *p* ≤ 0.05.

**Table 3:** Viral titer and morphological changes in the lung tissue of IVA-infected C57Bl/6 mice without treatment and after isRNA administration.

	Viral Titer, Lg FFU/mL	Vv Normal Lung Tissue, %	Vv Inflammatory Infiltration, %	Vv Blood Vessels, %	Nv Bronchi, %
Healthy	-	91.5 ± 1.4	-	3.6 ± 0.8	5.0 ± 0.7
**Day 1**					
Untreated	2.9 ± 0.7	79 ± 2 *	12.7 ± 1.4	2.1 ± 0.6 *	6.3 ± 0.2 *
MOCK	2.7 ± 0.7	83.7 ± 2.2 *^#^	8.8 ± 1.9 ^#^	2.1 ± 0.1 *	5.5 ± 0.2 ^#^
isRNA	1.2 ± 0.1 ^#^	92.2 ± 1.7 ^#^	2.7 ± 0.1 ^#^	2.2 ± 0.3 *	3.2 ± 0.7 *^#^
Cycloferon	1.2 ± 0.1 ^#^	91.1 ± 0.6 ^#^	2.1 ± 0.6 ^#^	2.7 ± 0.9	4.3 ± 0.3 ^#^
Day 3					
Untreated	3.8 ± 0.4	78.9 ± 3.1 *	16.8 ± 1.8	1.5 ± 0.5 *	2.9 ± 0.9 *
MOCK	4.0 ± 0.3	78.9 ± 4.5 *	12.2 ± 3.3	3.7 ± 0.9 ^#^	5.2 ± 0.4 *^#^
isRNA	4.2 ± 0.3	89.9 ± 1.2 ^#^	3.2 ± 0.5 ^#^	1.4 ± 0.4 *	5.5 ± 0.5 ^#^
Cycloferon	3.6 ± 0.5	89.8 ± 0.7 ^#^	5.2 ± 0.6 ^#^	1.3 ± 0.3 *	3.7 ± 0.3 *
Day 5					
Untreated	5.1 ± 0.6	67.8 ± 4.7 *	21.9 ± 4.6	4.8 ± 0.9 *	5 ± 0.5
MOCK	4.9 ± 0.6	66.8 ± 3.8 *	19.7 ± 2.5	5.1 ± 0.9	8.4 ± 1.3 *^#^
isRNA	4.6 ± 0.4	85 ± 0.8 *^#^	2.3 ± 0.6 ^#^	5.6 ± 0.4 *	7.5 ± 0.5 *^#^
Cycloferon	4.4 ± 0.3	84.1 ± 1.5 *^#^	3.7 ± 0.7 ^#^	5.5 ± 1.2	6.6 ± 1 ^#^

* Statistically significant difference relative to healthy C57Bl/6 mice, *p* ≤ 0.05. ^#^ Statistically significant difference relative to untreated IVA-infected C57Bl/6 mice, *p* ≤ 0.05.

**Table 4:** Immunohistochemical staining of lung tissue of IVA-infected C57Bl/6 mice without treatment and after isRNA administration.

	Healthy	Untreated	MOCK	isRNA	Cycloferon
TNF-α positive cells, Vv, %	14 ± 2.6	32.2 ± 4.7 *	34.2 ± 4.3 *	16.6 ± 3.2 ^#^	17.5 ± 2.7 ^#^
Lysozyme positive cells, Nv	26.3 ± 1	11.1 ± 0.6 *	13.4 ± 0.8 *	24.7 ± 1.1 ^#^	21.5 ± 0.9 ^#^

* Statistically significant difference relative to uninfected C57Bl/6 mice, *p* ≤ 0.05. ^#^ Statistically significant difference relative to untreated IVA-infected C57Bl/6 mice, *p* ≤ 0.05.
